# β-Glucan and Fatty Acid Based Mucoadhesive Carrier for Gastrointestinal Tract Specific Local and Sustained Drug Delivery

**DOI:** 10.3390/biom13050768

**Published:** 2023-04-28

**Authors:** Stephanie Vargas Esquivel, Himanshu N. Bhatt, Rimpy Diwan, Ahsan Habib, Wen-Yee Lee, Zehedina Khatun, Md Nurunnabi

**Affiliations:** 1 Aerospace Center (cSETR), University of Texas at El Paso, El Paso, TX 79968, USA; 2 Department of Aerospace & Mechanical Engineering, University of Texas at El Paso, El Paso, TX 79968, USA; 3 Department of Pharmaceutical Sciences, School of Pharmacy, University of Texas at El Paso, El Paso, TX 79902, USA; 4 Biomedical Engineering Program, College of Engineering, University of Texas at El Paso, El Paso, TX 79968, USA; 5 Department of Chemistry and Biochemistry, College of Science, University of Texas at El Paso, El Paso, TX 79968, USA

**Keywords:** mucoadhesive, β-glucan, oral medicine, local delivery, stomach specific

## Abstract

The oral route is considered the most convenient route of drug administration for both systemic and local delivery. Besides stability and transportation, another unmet but important issue regarding oral medication is retention duration within the specific region of the gastrointestinal (GI) tract. We hypothesize that an oral vehicle that can adhere and maintain retention within the stomach for a longer duration can be more effective to treat stomach-related diseases. Therefore, in this project, we developed a carrier that is highly specific to the stomach and maintains its retention for a longer duration. We developed a vehicle composed of β-**G**lucan **A**nd **D**ocosahexaenoic **A**cid (**GADA**) to observe its affinity and specificity to the stomach. GADA forms a spherical-shaped particle with negative zeta potential values that vary based on the feed ratio of docosahexaenoic acid. Docosahexaenoic acid is an omega-3 fatty acid that has transporters and receptors throughout the GI tract, such as CD36, plasma membrane-associated fatty acid-binding protein (FABP (pm)), and a family of fatty acid transport proteins (FATP1-6). The in vitro studies and characterization data showed that GADA has the capability to carry a payload of hydrophobic molecules and specifically deliver the payload to the GI tract, exert its therapeutic effects, and help to maintain stability for more than 12 h in the gastric and intestinal fluid. The particle size and surface plasmon resonance (SPR) data showed that GADA has a strong binding affinity with mucin in the presence of simulated gastric fluids. We observed a comparatively higher drug release of lidocaine in gastric juice than that in intestinal fluids, demonstrating the influence of the pH values of the media on drug-release kinetics. In vivo and ex vivo imaging of mice demonstrated that GADA maintains its retention within the stomach for at least 4 hr. This stomach-specific oral vehicle holds strong promise to translate various injectable therapeutic drugs to oral form upon further optimizations.

## 1. Introduction

The advancement of biotechnology has had a mass contribution to human health by the mass production of therapeutic macromolecules, including proteins, peptides, hormones, antibodies, and RNAs [1,2,3,4]. However, due to extensive biological barriers and the harsh gastric environment, most of the biological therapeutics (BT) are restricted to oral administration [5,6,7,8,9]. Due to sophisticated and sensitive properties, the BTs are denatured or protonated, and their higher molecular weights inhibit diffusion and transportation via the intestinal membrane [10,11,12]. Oral routes possess the advantages of patient compliance, convenience, and cost effectiveness, which make them favorable and accepted by patients of any age, gender, and race [13,14,15,16]. Therefore, oral is the most preferrable route for drug administration compared to the other routes, such as intravenous, subcutaneous, intramuscular, sublingual, rectal, nasal, and topical [17]. Despite tremendous demand and efforts, only ~50% of the therapeutic modalities are available in oral dosage forms [13]. Therefore, there is a need to design and develop formulations that can protect the macromolecules in the destructive GI environment and, thereby, increase the absorption and permeation in the stomach or intestine. It is reported that molecules with higher molecular weights (>500 Da) possess low solubility and absorption/permeation and, hence, show low bioavailability [18]. In addition, macromolecules also show poor absorption and low bioavailability due to their large molecular weight and higher lipophilicity or hydrophobicity. However, there have been various approaches explored by delivery scientists to overcome the barriers in oral drug delivery. For instance, chitosan is an emerging mucoadhesive carrier for the oral delivery of small and large molecules [19,20,21], ionic liquid is a promising carrier for the oral delivery of peptides [22,23,24], and various polymers are used for particle-based oral drug delivery [25,26,27]. Along the same line, we have explored the potential of bile acid transporter-mediated oral delivery of small molecules [28] and large molecules [29].

Site-specific oral delivery of therapeutics to treat local diseases within the gastrointestinal (GI) tract, specifically the stomach, is very challenging because of the lower retention time of an orally administered therapeutic, which varies from 15 to 30 min depending on a fasting or fed condition [30,31]. To improve the therapeutic outcomes for a local disease specific to the GI tract, such as stomach cancer, inflammatory bowel disease, and colon cancer, a longer retention of the therapeutic regimens is necessary. Over the years, to overcome the limitations of the oral delivery of therapeutics, different approaches have been taken, and among them, a targeting-based drug delivery approach shows better effects. “It promotes the slow release of drug in the stomach, which subsequently extends the time available for drug dissolution and absorption in the stomach and/or small intestine [7,32,33]”. However, there is still a lack of development of optimized formulations/vehicles that can be specifically delivered to the region of interest within the GI tract and exert their therapeutic effects for a longer duration [32].

β-glucans (BG) are a type of polysaccharide with a basic skeleton of β-(1,3)-glycosidic bonds that consist of glucose residues joined by beta linkages. It is a food additive approved by the U.S. Food and Drug Administration and considered safe [34]. β-glucans are also found to be effective for stimulation of the immune system and as cholesterol lowering agents [35,36]. In our previous study, we observed that β-glucan is very effective for the oral delivery of protein [37] and has stronger affinity and interaction kinetics with fatty acids, for instance docosahexaenoic acid (DHA) [38]. Our findings demonstrated that β-glucan and DHA form micelles in aqueous solution via electrostatic ionic interaction. DHA not only helps to form self-assembled mucoadhesive vehicles but also facilitates transportation of the payload via active diffusion through the intestinal membrane [32,39]. According to several previously reported research studies, the health benefits of β-glucan are extensive, including its potential to treat diabetes and associated cardiovascular diseases besides improving oral absorption [34,40,41]. The main advantage of β-glucans for drug delivery is a high stability in the harsh gastric environment that helps in drug transportation through the intestine, and hence, β-glucan can be used as a shielding material to protect the peptide, protein, antibody, and hormone [35,38]. We also investigated the potential of β-glucan as an oral drug delivery vehicle [32]. Many studies show that the presence of β-glucans in the digestive tract aid in the absorption of molecules [42].

Docosahexaenoic acid (DHA) is an omega-3 fatty acid that helps in drug transport, lowering triglycerides and blood pressure, reducing blood clotting, and decreasing the risk of heart failure [43]. In addition, research shows that DHA has therapeutic effects in the treatment of arthritis, atherosclerosis, depression, myocardial infarction, thrombosis, and some cancers [44,45,46]. The fatty acid transporters are present throughout the GI tract, including the stomach and small intestines; therefore, incorporation of DHA with β-glucan is expected to enhance the absorption, diffusion, and transportation of therapeutic payloads.

Therefore, in this project, our main objective was to prepare a drug delivery carrier, which can deliver the therapeutic payload specifically to the stomach and facilitate sustained release of the payload. β-glucan was used as a shielding material, and our developed vehicle shows a higher interaction with mucin in the gastric pH environment, as it enables vehicles a longer residency and higher potency in the stomach. The SPR (surface plasmon resonance) and High-Performance Liquid Chromatography (HPLC) data showed a high binding affinity and sustained release of the model drug lidocaine entrapped in GADA particles with higher stability in the GI tract environment. In vivo and ex vivo images of the mice orally administered with the GADA vehicle show a minimum 4 h retention within the stomach. Therefore, GADA holds a huge potential as an emerging vehicle for facilitating the oral delivery of a wide range of therapeutic modalities from small to large molecules.

## 2. Materials and Methods

### 2.1. Materials

β-glucan (m.w. 179,000) obtained from barley (low viscosity) was purchased from Megazyme (Ireland). DHA (m.w. 328.57) was purchased from Nu-Check Prep, Inc. (Elysian, MN, USA). Simulated gastric fluid with 0.2% NaCl in 0.7% (*v*/*v*) HCl, Acetate Buffer (1 M, pH 4.0), N-(3-Dimethylaminopropyl)-N-ethylcarbodiimide hydrochloride (EDC), N-Hydroxysuccinimide (NHS), and simulated intestinal fluid were purchased from Thermo Fisher Scientific (Waltham, MA, USA). Mucin from porcine stomach type II was purchased from Sigma Aldrich (Burlington, MA, USA). 1 M Glycine–HCl Buffer, pH 2.7 ± 0.1, 10X Concentrate was purchased from Polysciences, Inc. (Warrington, PA, USA). DiR (DiIC18(7); 1,1′-dioctadecyl-3,3,3′,3′-tetramethylindotricarbocyanine iodide) was purchased from Perkin Elmer (Waltham, MA, USA).

### 2.2. Preparation and Characterization of GADA

GADA particles were prepared using different ratios of β-glucan and DHA individually and in combination to observe the interaction with mucin, gastric fluid, intestinal fluid, and PBS. Briefly, GADA was prepared by dissolving 2 mg of β-glucan in 1 mL of either PBS, intestinal fluid, or gastric fluid and constantly heated and stirred at 70 °C for 15 min or until the solution became clear. Then, different volumes of DHA (2.5, 5, 10, 20, and 40 µL) were added into the clear β-glucan (2 mg) solution, and each combination was stirred for 30 s. For the next samples, 2 mg of mucin was added into the GADA particles composed of different ratios of β-glucan and DHA to assess the interaction of GADA with mucin. For comparison, different amounts of mucin (2, 1, 0.5, 0.25, and 0.125 mg) were added into the β-glucan solution. The microparticles were observed for particle size and zeta potential determination with the dynamic light scattering technique.

### 2.3. Size and Morphology

The particle size and zeta potential were measured using a dynamic light scattering (DLS) instrument (Malvern Instruments Ltd., Zetasizer-nano ZS series, Malvern, UK) [47]. To measure the particle size and zeta potential of the vehicle, 1 mg/mL of GADA was dissolved in distilled water (DW), then the solutions were taken in a disposable folded capillary cell suitable for light scattering. To assess the morphology of the prepared formulations, a scanning electronic microscope (SEM) was used. The freshly prepared samples were placed on a copper grid with a carbon film, placed in an oven to dry, and then kept at room temperature for 15 min before analysis [48].

### 2.4. Surface Plasmon Resonance (SPR)

All of the above samples were characterized using SPR to evaluate the interaction of the microparticles with diverse quantities of mucin (1, 2, 3, 4, and 5 mg) in different fluids, such as simulated gastric juice, intestinal fluid, and PBS. A total of 0.4 mL of EDC-NHS, 50 mL of PBS, 2 mL of glycine diluted in DW, and 1 mL of the above microparticles were used for the SPR measurement. All measurements were carried out at room temperature. The surface plasmon resonance (SPR) was measured using the iMSPR series from iClubio (Seoul, Republic of Korea).

### 2.5. Drug Loading and In Vitro Release Testing

Lidocaine was used as the model drug to determine the release from the GADA particles. Lidocaine was loaded in the GADA particles with the incubation method. Briefly, 2 mg of β-glucan was dissolved by constant heat and stirring for 15 min in 1 mL of either gastric juice or intestinal fluid. Once the samples turned transparent, different amounts of DHA (40, 20, 10, 5, and 2.5 mL) were added into the sample. Next, 10 mg of lidocaine was added to the samples; then, the samples were vortexed for 1 min to mix properly and kept at room temperature for an 8 hr incubation. Then, the solutions were centrifuged 3 times at 10,000 RPM for 10 min to obtain the pellet of lidocaine-loaded microparticles and to remove the unreacted or free lidocaine. A direct method of estimation was used for the quantification of lidocaine in the prepared formulation. The obtained pellet was resuspended in 2 mL of acetonitrile, and the resultant suspension was centrifuged at 12,000 RPM for 15 min to completely extract the encapsulated lidocaine from the submicron particles. The supernatant was collected and injected into the HPLC for the quantification.

The release studies were performed in triplicate by using a dialysis membrane (MWCO 500 Da). After centrifugation, the resuspended particles (either in gastric juice or intestinal fluid) were filled into a dialysis bag, and then, the dialysis bag was immersed into 100 mL of the release medium at 37 °C (simulated gastric juice or simulated intestinal fluid) with a magnetic stirrer stirring at 100 rpm. One mL of the samples was withdrawn at different time points *viz*., 5, 10, 15, 30 min and then 1, 2, 3, 4, 6, 8, 10, 12, 18, and 24 h, and the same volume was replaced either with simulated gastric juice or intestinal fluid.

The amount of lidocaine released was determined by using HPLC Waters (Medford, MA, USA) using column XTerra RP18 4.6 × 150 mm, 5 μm. The mobile phase was H_2_O/ACN/100 Mm NH_4_COOH, pH 7.0 with a solvent ratio of 55:35:10 *v*/*v*/*v* and a flow rate of 1 mL/min at λmax 238 nm. The experiment was performed in triplicate. The graph was plotted for the percentage cumulative amount of lidocaine released versus time (mean ± SD, *n* = 3).

### 2.6. In Vivo and Ex Vivo Imaging

To investigate the in vivo and ex vivo GI tract retention of orally administered GADA, fluorescence dye, DiR (Excitation/Emission: 748/780 nm), was loaded into the vehicle and orally administered to the mice. The mice were anesthetized and then imaged using an in vivo imaging system (IVIS) at different time points of pre- and post-administration, according to the method published earlier [49]. For the ex vivo imaging, the mice were sacrificed after 8 h of post-administration, and the GI tract was harvested.

### 2.7. Statistics

All the experiments in this paper were performed in triplicate, and the data are presented as the mean ± standard deviation. The statistical significance was evaluated with the one-way ANOVA test using Graph Pad prism 8 (GraphPad Software, Inc., Boston, MA, USA). The data were considered statistically significant when the *p*-value < 0.05.

## 3. Results

### 3.1. Synthesis and Characterization

**Figure 1:**
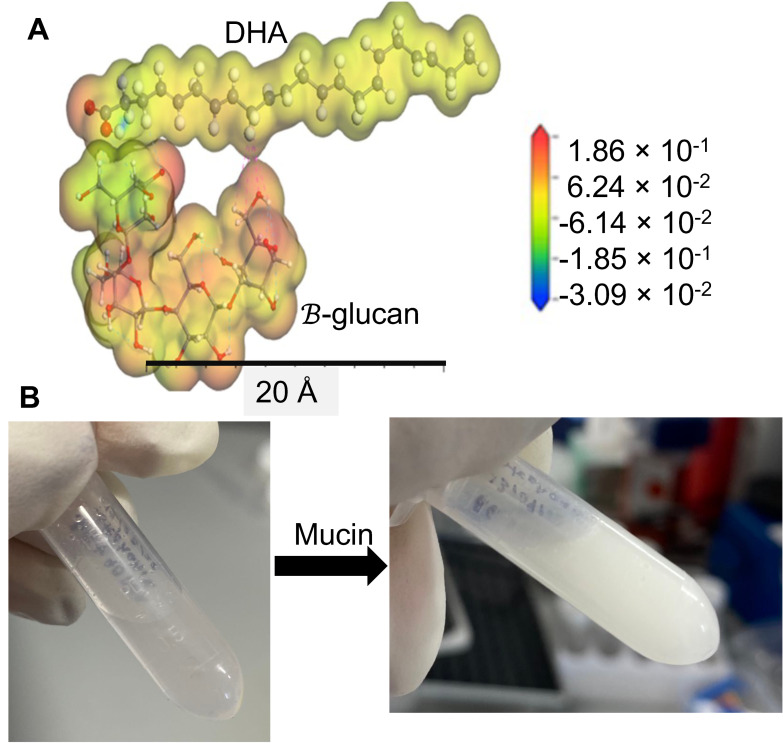
Chemical and physical properties. The molecular docking shows the ionic interaction between β-glucan and DHA in the presence of water (**A**). The GADA solution turns cloudy once mucin is added because of the interaction and aggregation, an indication of crystallization (**B**).

β-glucan and different amounts of DHA were physically mixed to prepare the GADA, which produced micron-sized particles via ionic interaction as shown in Figure 1A. Previously reported data showed that microparticles give a vehicle some excellent properties, including higher stability and catalytic activity [38]. Once the GADA was prepared, the mucin solutions were added to observe the interaction between mucin and GADA and elucidate the ability of GADA to interact and bind with mucin (major component in the gut and gut layer). Mucin helps to protect the intestine and tissue lining from corrosive acidic gastric juice, but mucin also prevents the transportation and diffusion of orally administered therapeutic molecules [50,51]. Figure 1B shows that the interaction between the GADA solutions and mucin was very prompt, and the interaction was self-facilitated and did not require any catalyst, temperature, or even longer duration. The GADA solution turned cloudy immediately after addition of the mucin due to aggregation of the microparticles. This is an indication of self-assembled microparticle formation in the presence of the aqueous solution.

**Figure 2:**
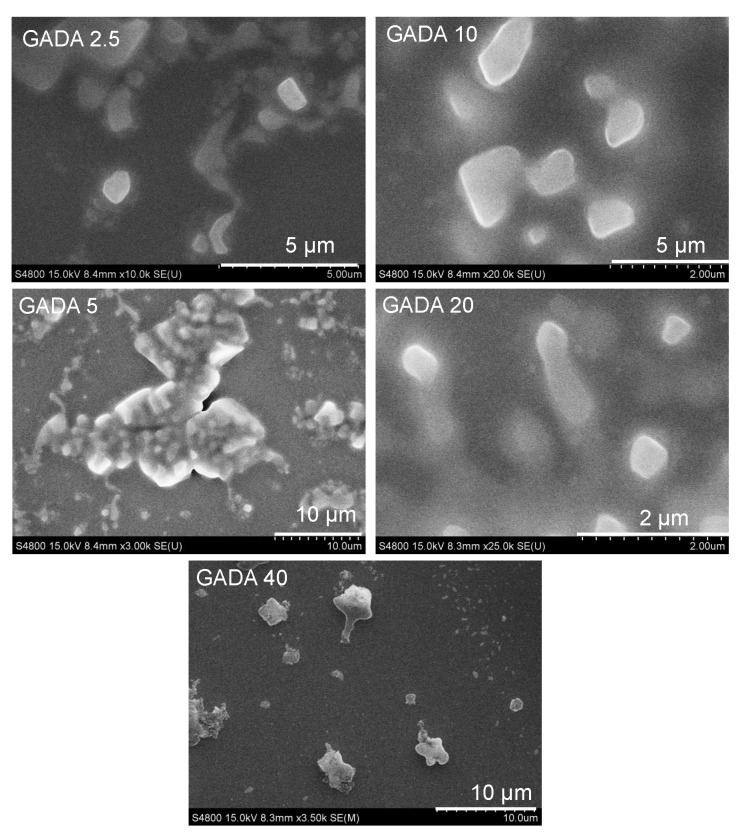
Size and morphology of the GADA derivatives observed with a scanning electron microscope (SEM).

Figure 2 shows the size and morphology of GADA by SEM. The images of the GADA particles show a nearly spherical shape without visible pores on the surface. The size of GADA varied from 2 to 10 mm depending on the concentration of DHA that was added with each mole of β-glucan. The overall characterization results indicate that β-glucan and DHA form self-assembled ionic particles that have a strong affinity with mucin, which is an indication of their mucoadhesive property. While most of the formulation shows some variation in their size, all the particles are approximately 1–2 mm in diameter as shown in the SEM images.

### 3.2. Interaction between GADA and Mucin

**Figure 3:**
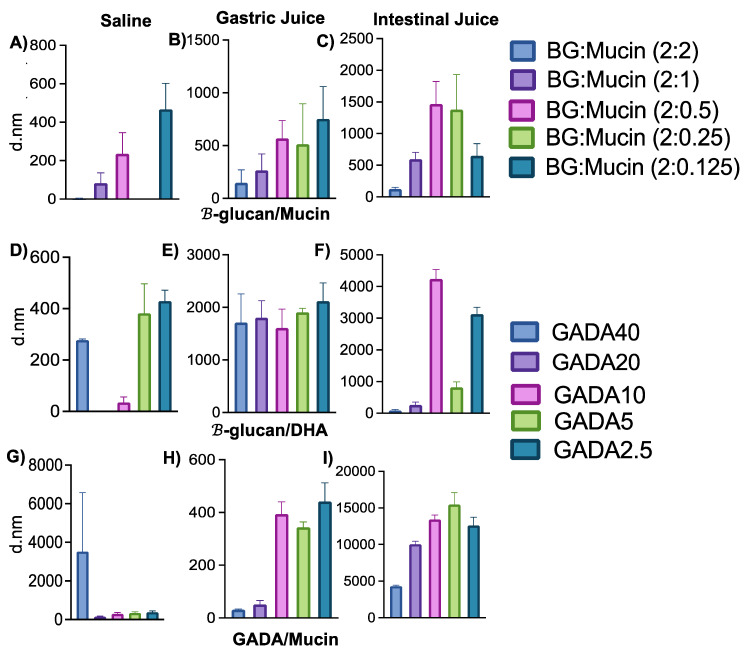
The graphs represent the particle size of each composite containing different amounts of either β-glucan, DHA, or mucin. (**A**) β-glucan with mucin in PBS. (**B**) β-glucan with mucin in gastric juice. (**C**) β-glucan with mucin in intestinal fluid. (**D**) GADA solution in PBS. (**E**) GADA solution in gastric juice. (**F**) GADA solution in intestinal fluid. (**G**) GADA with mucin in PBS. (**H**) GADA with mucin in gastric juice. (**I**) GADA with mucin in intestinal fluid. Data are shown as the mean ± SD (*n* = 3).

To investigate the effect of saline, gastric juice, and intestinal fluid on GADA and the interaction between GADA and mucin, we investigated their particle formation upon addition of these individual simulated fluids. We observed particle formation of BG with mucin, GADA, and GADA/Mucin in the presence of PBS (pH 7.4), simulated gastric juice (GJ), and intestinal fluid (IF). This data helped us to interpret the fate of GADA within the stomach and small intestine. Figure 2 shows the particle size of each formulation in the presence of mucin. Figure 3A–C shows that β-glucan itself forms particles in the presence of mucin. The particle size of β-glucan in mucin (2 mg; same amount of mucin was used for all the samples) in PBS (Figure 3A), gastric juice (Figure 3B), and intestinal fluid (Figure 3C) was measured with dynamic light scattering (DLS). The particle size of β-glucan/Mucin in saline, GJ, and IJ is highly corelated with the concentration of mucin. The size of β-glucan decreased as the concentration of mucin increased, regardless of the solvent. This is an indication of a higher ionic interaction between the two individual components, and the interaction forces are increased when mucin is increased. We also investigated how saline, GJ, and IJ effect the formation of GADA particles (Figure 3D–F). We observed similar trends in GADA in the presence of saline, but interestingly, we observed that gastric juice, which is acidic (pH 4), had no effect on the GADA particles. With an increase or decrease in DHA, the size of GADA was very much consistent. The particle size of GADA in the intestinal juice (pH 9) shows a huge increase in the size up to 4000 nm (GADA10). Interestingly, when GADA was added to the mucin in the presence of PBS, the GADA40 shows an increase in size of approximately 3500 nm, where the other four ratios show a stable size (Figure 3G). Figure 3H shows a huge increase in the particle size of the vehicle with mucin in gastric juice, where we observe that the vehicle particle size is stable in mucin with intestinal fluid (Figure 3I). From the size data, we observe that mucin with PBS shows size stability of both β-glucan and the vehicle. The particle size of the vehicle drastically changes when added with gastric juice and intestinal fluid solutions with or without mucin, which suggests that there is no correlation between the concentration of mucin, gastric juice, and intestinal fluid, but there might be a correlation between the size and concentration of DHA. In addition, our observation suggests that the GADA particles form an aggregation in the presence of mucin in the solution.

**Figure 4:**
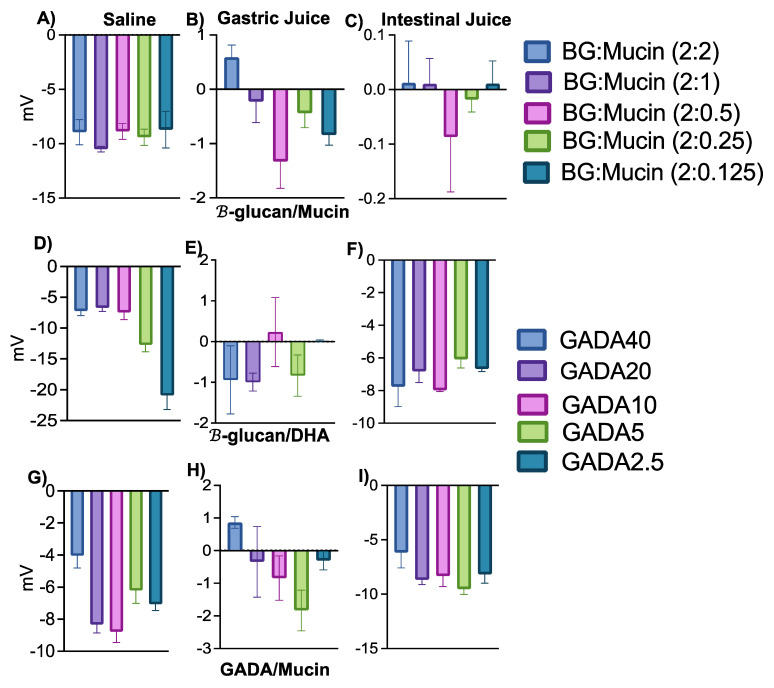
The zeta potential values for each matrix having different amounts of β-glucan, DHA, or mucin. (**A**) β-glucan with different concentrations of mucin (2, 1, 0.5, 0.25, 0.125 mg) in PBS. (**B**) β-glucan with different concentrations of mucin (2, 1, 0.5, 0.25, 0.125 mg) in gastric juice. (**C**) β-glucan with different concentrations of mucin (2, 1, 0.5, 0.25, 0.125 mg) in intestinal fluid. (**D**) GADA solution in PBS. (**E**) GADA solution in gastric juice. (**F**) GADA solution in intestinal fluid. (**G**) GADA with mucin (2 mg) in PBS. (**H**) GADA with mucin (2 mg) in gastric juice. (**I**) GADA with mucin (2 mg) in intestinal fluid. Data are shown as the mean ± SD (*n* = 3).

β-glucan is a slightly positively charged polysaccharide. However, the surface charge of β-glucan/Mucin was measured as −10 mV when dissolved in saline, which is a result of the deposition of the negatively charged mucin on the surface of β-glucan (Figure 4A). Though most of the β-glucan/mucin composition shows a negative surface charge while dissolved in gastric juice, the same formulations show a partial positive charge while dissolved in simulated intestinal juice (Figure 4B,C) depending on the concentration of mucin.

The GADA formulations show different trends than β-glucan. Regardless of whether the solvent is saline, gastric juice, or intestinal fluid, GADA always shows negatively charged surface charges. For GADA with mucin in gastric juice, the zeta potential values went from highly negative to less negative depending on the concentration of DHA, except for the highest concentration of DHA, which showed positive values (Figure 4H). The same trend was observed with β-glucan and mucin in gastric juice (Figure 4B). Figure 4A,I shows the negative charge of GADA with mucin in gastric juice and intestinal fluid. Mucin itself has a negative charge of approximately −50 mV [52]. Therefore, from Figure 4, we can deduce that β-glucan and mucin do not impact the surface charge on GADA, but the DHA concentration might have effects on the zeta potential value.

**Figure 5:**
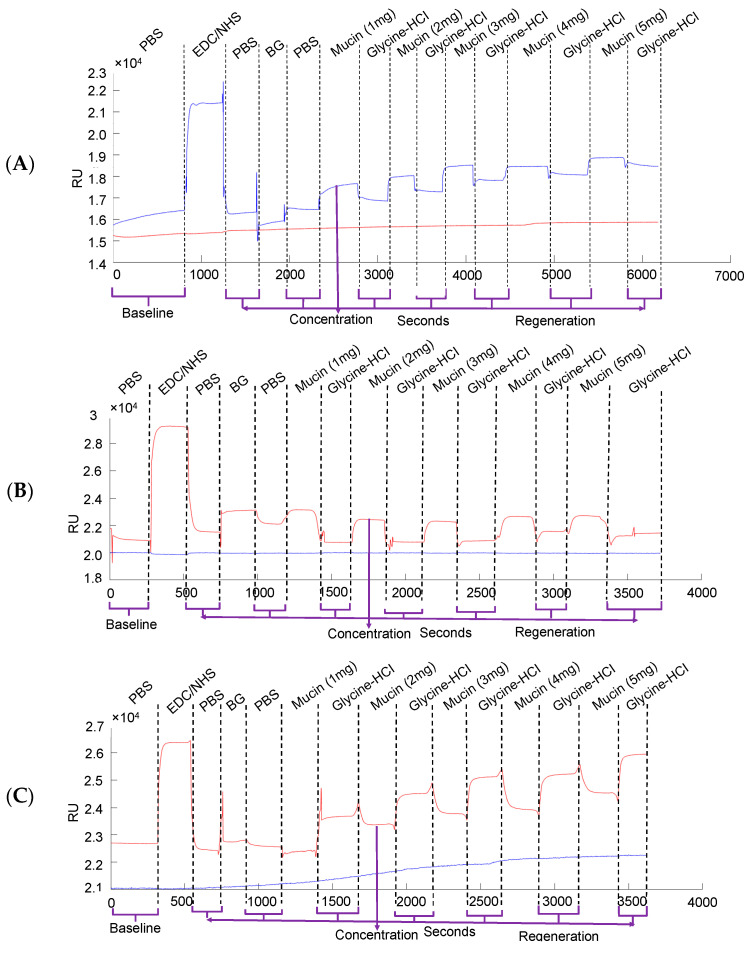
SPR analysis on the molecular interaction between β-glucan and different amounts of mucin in (**A**) saline, (**B**) gastric juice, and (**C**) intestinal juice.

The graphs in Figure 4 outline the molecular interaction between β-glucan and different amounts of mucin in solvents with variations in pH, such as saline, gastric juice, and intestinal juice. PBS was used as a control considering that the pH is near neutral values (7.4), while simulated gastric juice and intestinal juice have acidic and basic pH, respectively. EDC-NHS was used for a better attachment between the particles when in contact with the Au chip for SPR analysis [53]. Glycine–HCI was used as a regeneration buffer to quickly reverse the interaction between the analyte and ligand, allowing the functionalized sensor surface to be used again. The peaks show the attachment of mucin to the chip, which initially had β-glucan. The association and dissociation between the ligand and analyte were clearly observed, meaning the particles were attached and detached as expected. When the peak becomes flat, it means it has reached an equilibrium, also called the maximum concentration. Based on the graphs, it was observed that there is a direct relationship between the intensity of the signal and the amount of mucin. In Figure 5A,B, where the solvents were PBS and gastric juice, respectively, the peaks started to increase along with the amount of mucin; the highest signal was for 5 mg of mucin. When intestinal fluid was used, there was a more constant signal even though the mucin amount was increasing (Figure 5C). The findings demonstrate that β-glucan is very stable in acidic GJ and maintains its chemical and mucoadhesive properties. This is also an indication that β-glucan has even more binding affinity within the stomach, as the mucin concentration is higher in gastric juice than that of intestinal juice.

**Figure 6:**
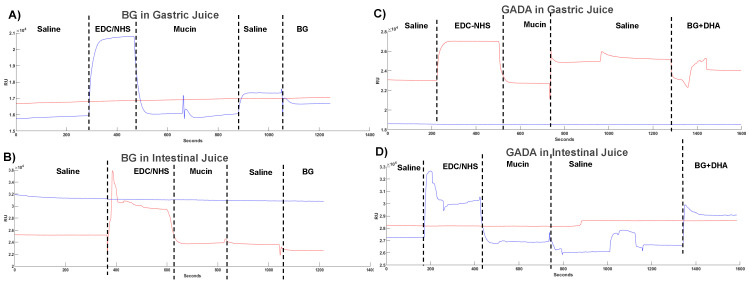
SPR shows the interaction of mucin with β-glucan in gastric juice (**A**) and intestinal juice (**B**), and GADA and mucin in gastric juice (**C**) and intestinal juice (**D**) to observe the influence of various solvents on the molecular interaction between GADA and mucin.

Figure 6 compares the molecular interaction between GADA and mucin in the presence of GJ and IJ and compares the interaction profile between β-glucan and mucin. To have a more accurate analysis, the ligand was mucin, and the analyte was either β-glucan or GADA to observe if there was an attachment with mucin located throughout the gastrointestinal tract. Based on the association and dissociation phases in all the graphs, we observe that there was indeed a higher binding affinity between GADA and mucin than between β-glucan and mucin. The signal was higher when GADA was used (Figure 6C,D) compared to β-glucan by itself (Figure 6A,B). Based on that, we assume GADA has better molecular attachment with mucin compared to β-glucan by itself in both gastric juice and intestinal fluid.

Again, comparing the interaction between GADA and mucin in GJ and IJ, we observe that GADA has more binding affinity with mucin when dissolved within GJ. The data indicate the ability of GADA and its binding affinity with stomach mucus.

### 3.3. Drug Loading Efficiency and Release Profile

**Figure 7:**
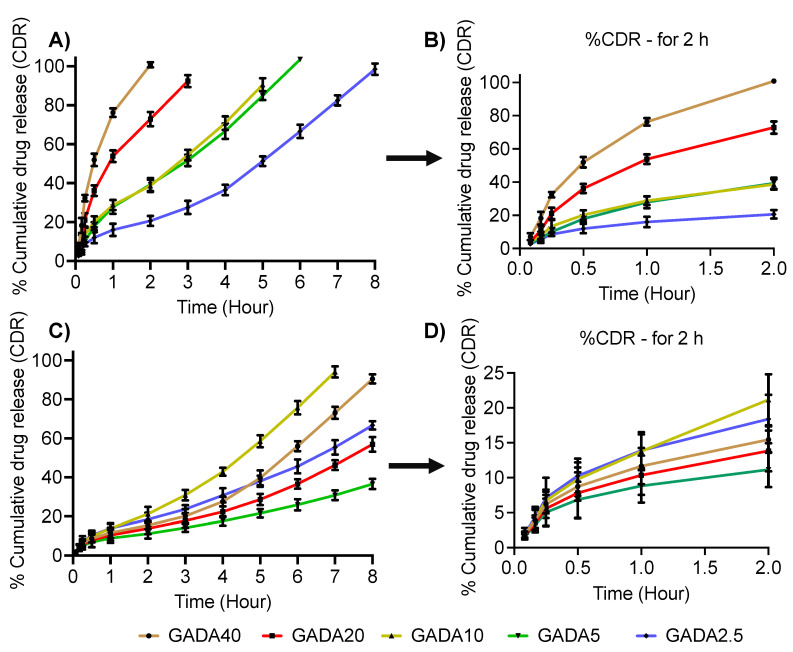
In vitro release profile of hydrophobic small molecules in gastric juice (**A**,**B**) and in intestinal fluid (**C**,**D**). The release profile demonstrates that a higher and prompt release profile was observed in gastric juice compared to that in intestinal fluid. Data are shown as the mean ± SD (*n* = 3).

To observe loading efficiency and release profile, we chose lidocaine, a hydrophobic small molecule, which is used as a local anesthetic [54]. The drug loading was found to be 5–8% in all the ratios of GADA. The in vitro release of lidocaine is shown in Figure 6. Figure 7B,D is the zoomed version (release up to 2 h) of Figure 7A,C. It is observed from Figure 7B that more than 90% of the drug release was observed in 120 min in the case of GADA40, while GADA2.5 showed more than 90% release in 8 h in gastric fluid. In contrast, it is observed from Figure 7C that more than 90% of the drug release was observed in 8 h in the case of GADA40, while GADA2.5 showed more than 90% release in 7 h in intestinal fluid. This finding confirms that β-glucan and DHA have more interaction in the gastric environment than in the intestinal environment. These results are in support of the SPR results. While the lidocaine release profile shows a burst release for most of the formulations, the same formulations show a sustained release trend in intestinal fluid. This indicates that an acidic environment facilitates the degradation of microparticles and, thereby, enhances release of the payload.

### 3.4. In Vivo Imaging and Biodistribution

**Figure 8:**
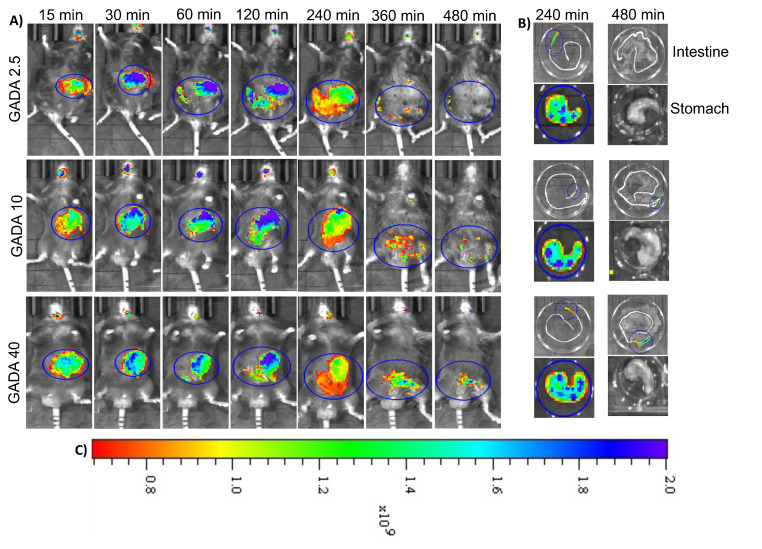
In vivo imaging of the developed GADA formulations given through an oral route at a DiR dose of 1 mg/kg. Orally administered GADA derivatives show that they are highly specific to the stomach and maintain retention in the stomach for 4–6 h (**A**). Blue indicated higher intensity and red indicates lesser intensity of the entrapped fluorescent dye DiR. Ex vivo imaging further confirms their retention in the stomach for such duration, which is significantly higher than the intestine (**B**). Different color within the images indicates the intensity of the fluorescence dye and their amount of localization within specific organs. Color-coded scale indicates the intensity based on accumulation of the fluorescent dye-loaded GADA (**C**).

To investigate the in vivo biodistribution and organ-specific localization of the orally administered GADA formulations, we incorporated fluorescence dye, DiR, within the GADA. The mice were imaged non-invasively using an in vivo imaging system (IVIS) upon oral administration. The in vivo images show that the GADA formulations were able to maintain their retention within the stomach for at least 4 h post-administration. Though all formulations were retained within the stomach for 4 h or more, GADA10 maintained a higher intensity up to 6 h, which is an indication of its ability to hold the payload and its higher mucoadhesiveness (Figure 8A). To confirm further, the GI tracts were harvested and isolated for ex vivo imaging (Figure 8B). The ex vivo images show that the GADA formulations are very specific to the stomach, as very nominal or no signal of the fluorescence dye was observed throughout the intestines. With this in vivo and ex vivo imaging, we confirm that GADA is a newly designed and developed oral drug delivery vehicle that is very specific to the stomach. It also indicates that GADA10 has a better ability to maintain sustained release of the hydrophobic payload within stomach environments and maintain its properties in the harsh gastric environment.

## 4. Conclusions

In this project, we developed a GI tract-specific, oral drug delivery vehicle and investigated its stability and binding affinity within the GI tract specific to the stomach and intestine. We also investigated its loading efficiency of hydrophobic small molecules and release profile in various mediums, including saline, acidic gastric juice, and basic pH of intestinal fluid. Our investigation and the data demonstrate that the fatty acid-linked oral vehicle has specificity to the fatty acid receptors, including CD36, plasma membrane-associated fatty acid-binding protein (FABP), and a family of fatty acid transport proteins (FATP1-6), which are overly expressed in the GI tract. The in vivo biodistribution and organ specificity proved the hypothesis, as the retention of the orally administered GADA was viable and visible for over 4 h post oral administration, observed in vivo and ex vivo. We observed the ability of GADA to orally deliver hydrophobic small molecules, which holds strong promise for biological therapeutic delivery. The GADA carrier can maintain retention of the orally administered payload for up to 4 h, which is much longer than many well-known drugs, for instance ranitidine and misoprostol that have gastrointestinal retentions of only 12 and 8 min, respectively. The payload release profile also demonstrates that the release of the hydrophobic drug lidocaine was controlled, but acidic gastric juice facilitated a faster release than intestinal fluid. Therefore, we further aim to investigate the potential of GADA for the delivery of oral biologics in an animal model.

## Data Availability

Not applicable.
